# Supplementary figures and images for: The Effects of Selective Inhibition of Histone Deacetylase 1 and 3 in Huntington’s Disease Mice
Source: Front Mol Neurosci. 2021 Feb 17;14:616886. doi: 10.3389/fnmol.2021.616886 (PMC7925995; doi:10.3389/fnmol.2021.616886)

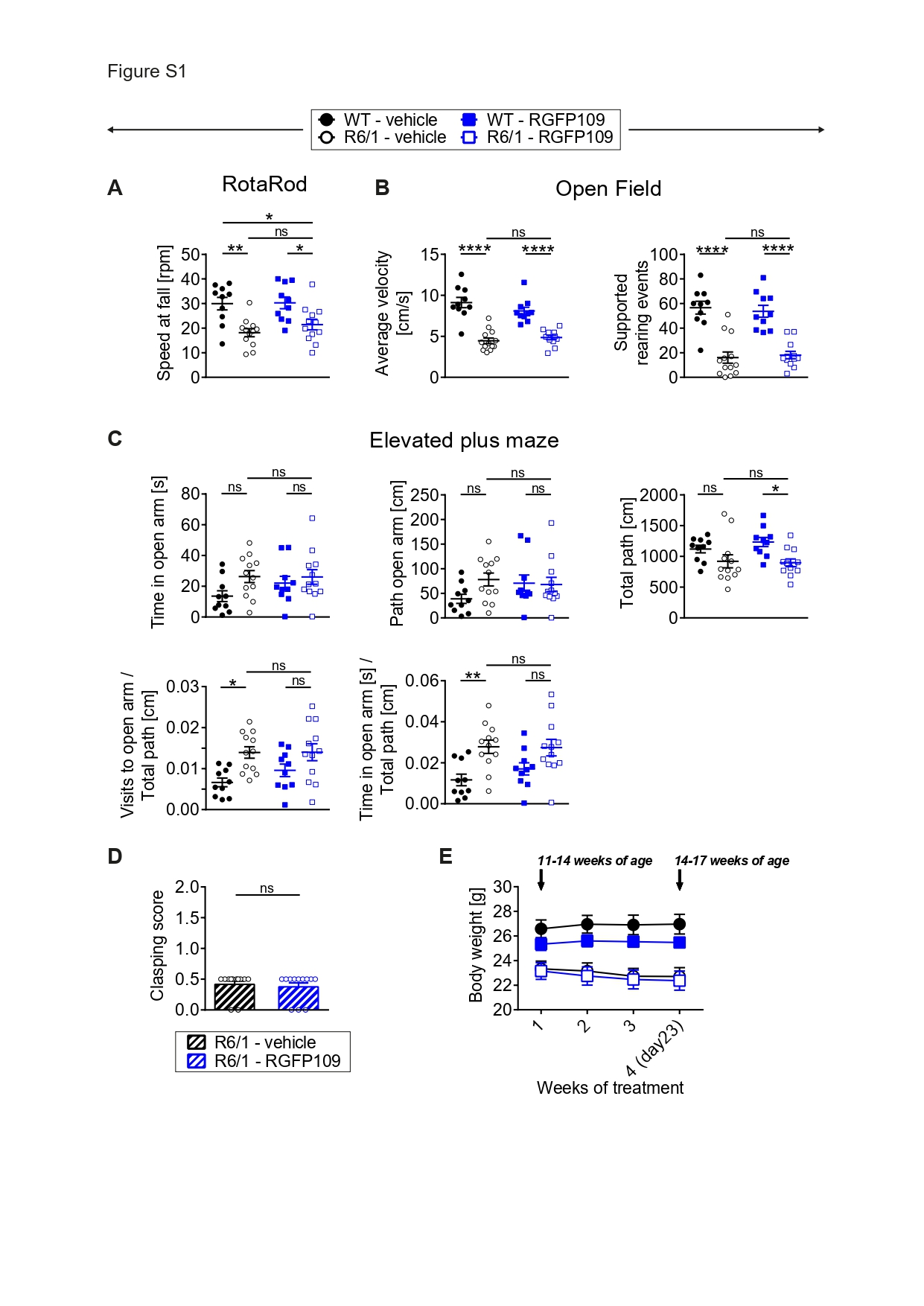

Supplement: Supplementary file 1 [file Image_1.jpg]

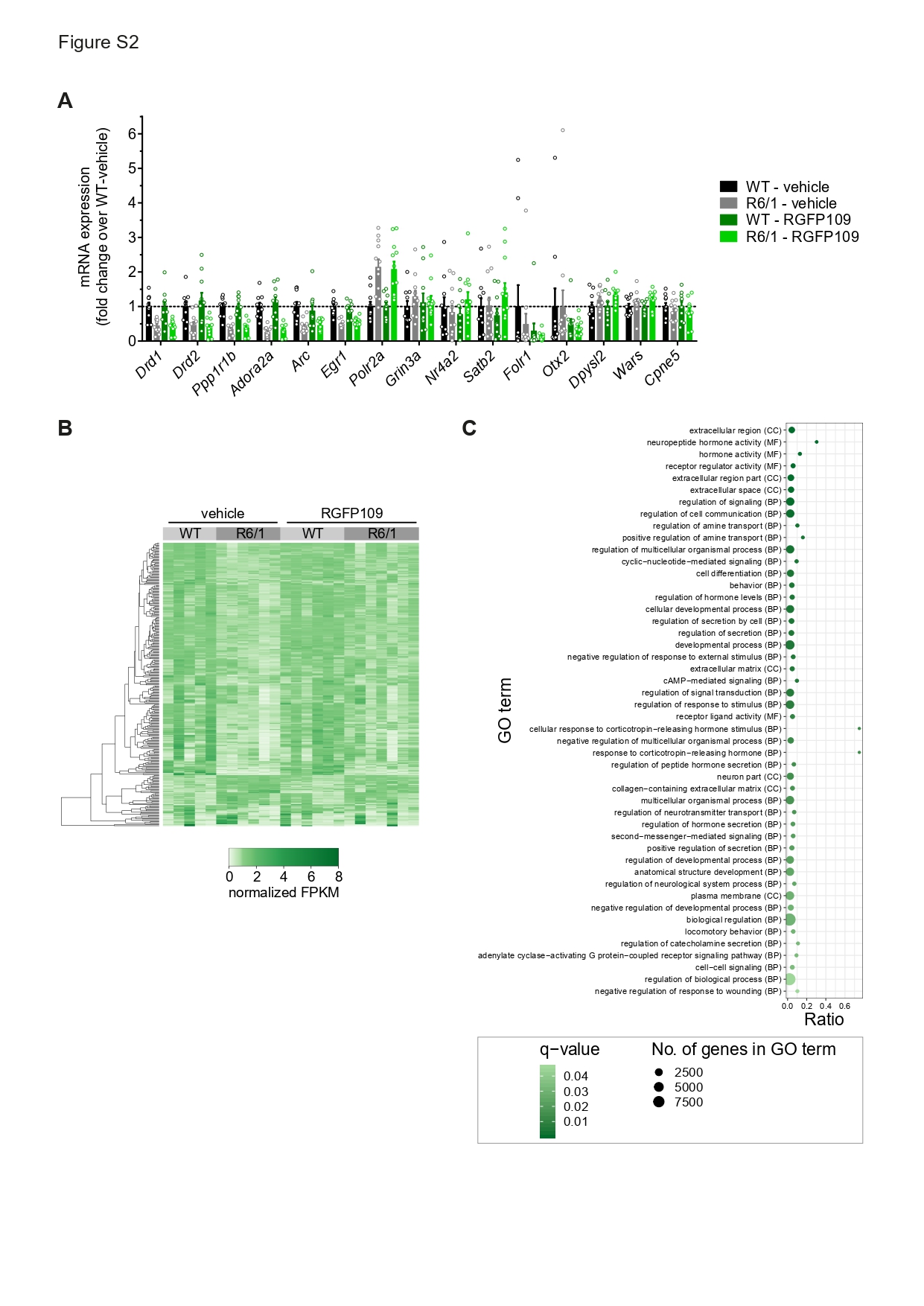

Supplement: Supplementary file 2 [file Image_2.jpg]

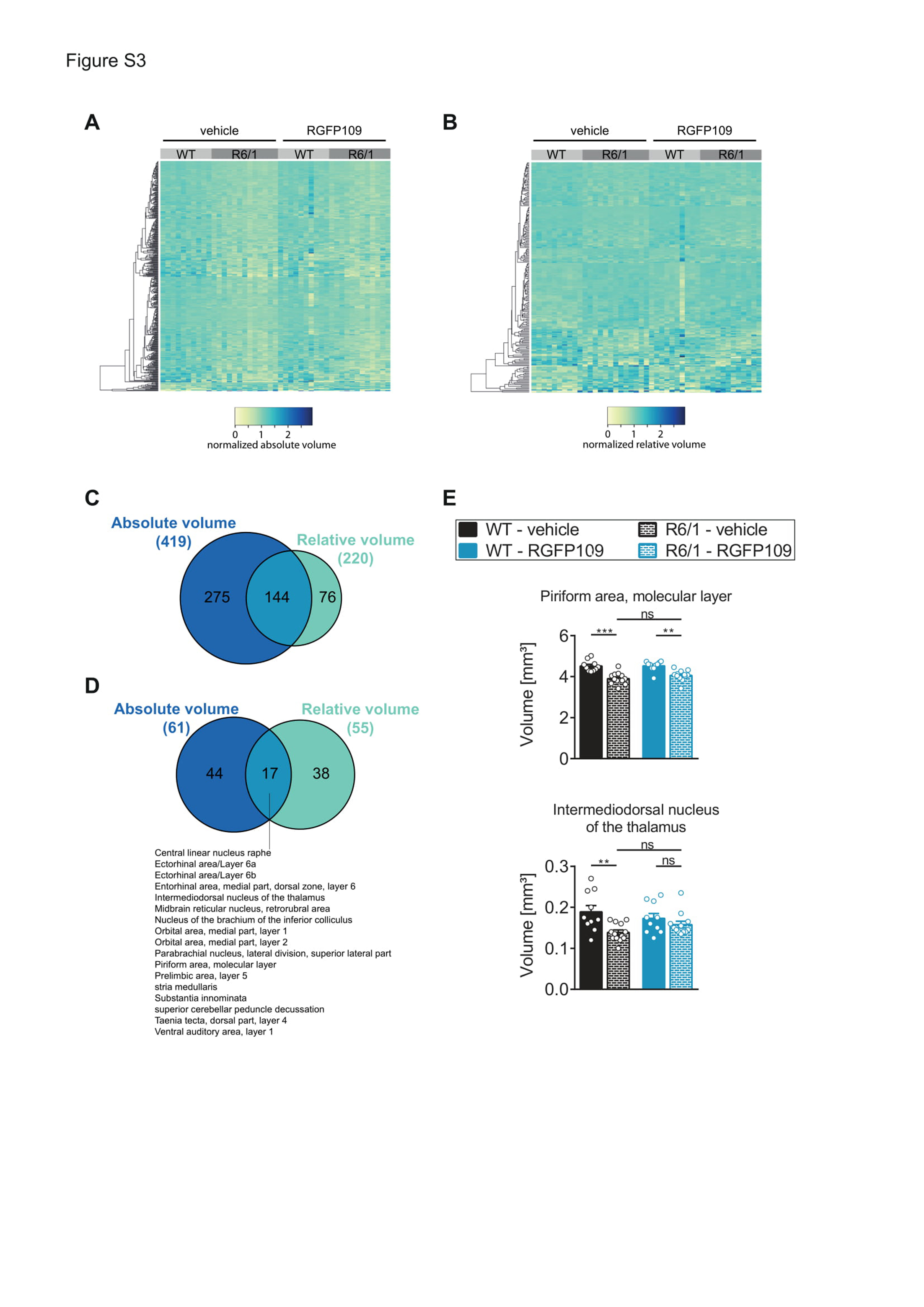

Supplement: Supplementary file 3 [file Image_3.jpg]

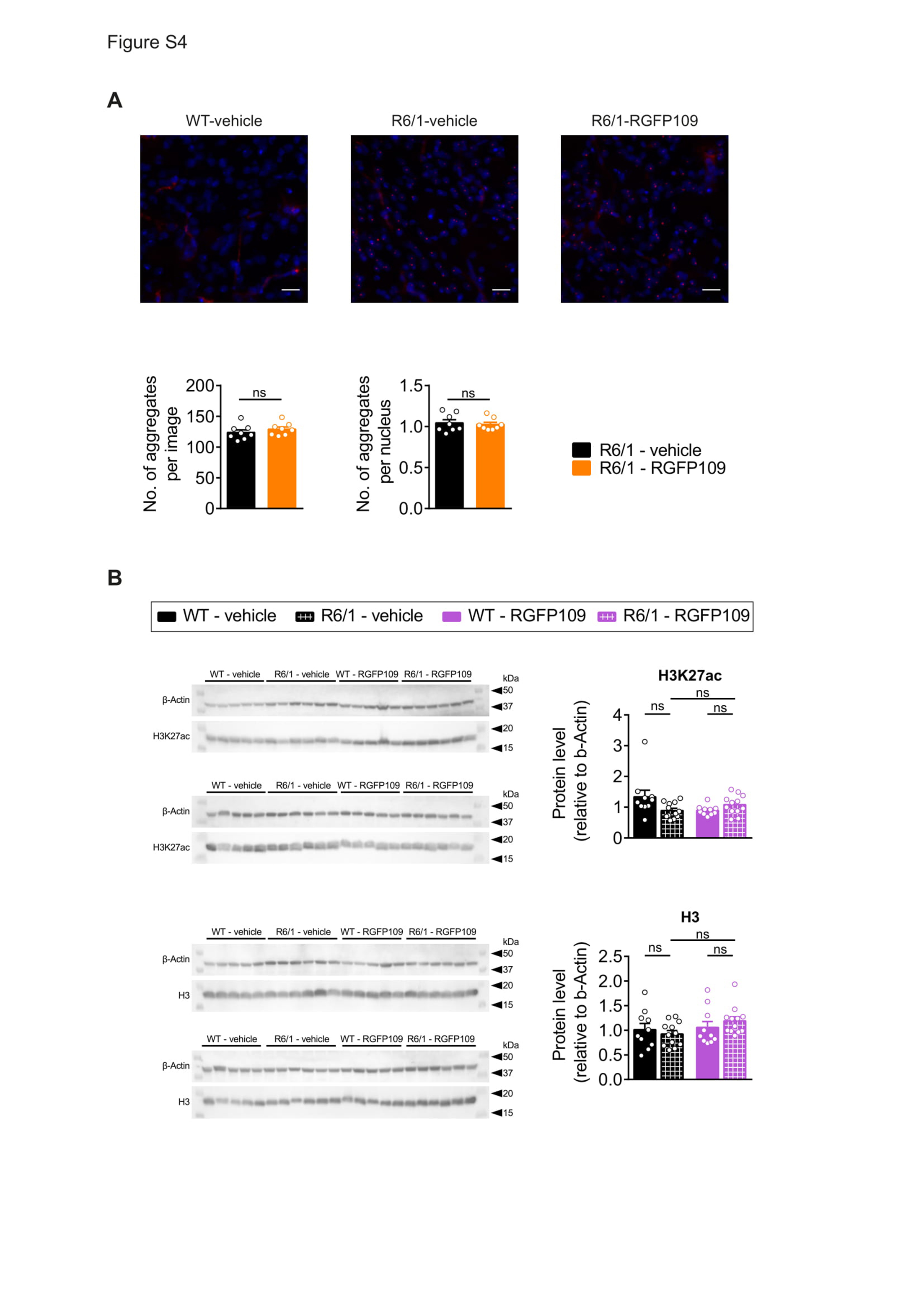

Supplement: Supplementary file 4 [file Image_4.jpg]
