# Supplementary material for: The Effects of Selective Inhibition of Histone Deacetylase 1 and 3 in Huntington’s Disease Mice
Source: Front Mol Neurosci. 2021 Feb 17;14:616886. doi: 10.3389/fnmol.2021.616886 (PMC7925995; doi:10.3389/fnmol.2021.616886)
Supplement: Supplementary file 5 [file Image_5.pdf]

Supplementary Figure Legends

Figure S1. Behavioral and physical assessment of vehicle- and RGFP109-treated wild type and R6/1 mice. (A) Motor performance on a RotaRod: Speed at fall as mean of all trials. (B) Open field exploration evaluating general locomotor activity measured as average velocity and vertical activity (supported rearing). (C) Elevated plus maze test showing time in open arm, path in open arm, total path, and visits and time in open arm normalized to total path. (D) Clasping score of R6/1 mice measured at the end of the study. (E) Body weight over the course of the treatment. Data presented as mean±SEM; n=10 [WT-vehicle], n=12(13, Open Field) [R6/1-vehicle], n=10 [WT-RGFP109], n=12 [R6/1-RGF109]; *p<0.05, **p<0.01, ***p<0.001, ****p<0.0001, ns - not significant by two-way ANOVA with Tukey‘s multiple comparisons test (A-C) or by unpaired Student's t-test (D).

Figure S2. Effect of HDAC1 and HDAC3 inhibition by RGFP109 on gene expression in striatum of R6/1 mice. (A) Quantitative real-time PCR analysis of genes usually up- and downregulated in HD, motor-learning associated genes and those genes whose expression are found to be significantly altered in RNA-seq data by RGFP109 treatment of R6/1s in the striatum of vehicle- and RGFP109-treated wild type (WT) and R6/1 mice. Data presented as mean±SEM; n=10 [WT-vehicle], n=12 [R6/1-vehicle], n=10 [WT-RGFP109], n=12 [R6/1-RGF109]. (B) Heatmap showing differentially expressed genes of R6/1 and WT comparison (FDR, q<0.1, log2 FC >|0.5|) which are changed towards WT levels by RGFP109 treatment in R6/1 mice by at least 20% (283). For better visualization FPKM values were mean-normalized. (C) Gene Ontology (GO) analysis of genes from (B). As background, all expressed genes with FPKM>0.1 in at least one sample were used (18844). Ratio describes the ratio between genes in the target gene list and the total number of input genes associated with a specific GO term. Enriched GO terms with FDR, q<0.05 are plotted; BP – biological process, CC – cellular component, MF – molecular function. n=5 [WT-vehicle], n=6 [R6/1-vehicle], n=6 [WT-RFGP109], n=7 [R6/1-RGFP109].

Figure S3. Influence of RGFP109 treatment on brain substructures in R6/1s measured by volumetric MRI. (A, B) Heatmaps illustrating absolute (A) or relative (B) volumes of regions with significant volume difference in WT-vehicle and R6/1-vehicle comparison (419 regions for absolute and 220 regions for relative volume). Volumes of every region were normalized to the mean volume of each region over all samples. (C) Venn diagram indicating shared and unique brain regions with significant volume change in R6/1 mice from absolute and relative volume analysis. (D) Venn diagram indicating shared and unique brain regions affected positively by the drug by at least 20% (not-significant) in R6/1 mice (red framed regions of Figure 3A and B) from absolute and relative volume analysis. (E) Absolute volumes of selected brain regions. Data presented as mean±SEM; n=10 [WT-vehicle], n=13 [R6/1-vehicle], n=10 [WT-RGFP109], n=12 [R6/1-RGFP109]; *q<0.05, **q<0.01, ***q<0.001, ****q<0.0001, ns - not significant.

Figure S4. Analysis of aggregate formation and bulk histone H3K27 acetylation levels in vehicle- and RGFP109-treated wild type and R6/1 mice. (A) Top: Representable images of immunohistochemical staining of HTT-aggregates (red, EM48) and nuclei (blue, DAPI) in striatal sections of vehicle-treated WT (left), vehicle-treated R6/1 (middle) or RGFP109-treated R6/1 mice (right). Scale bar = 20 µm. Bottom: Quantification of aggregate counts in R6/1 mice. Data presented as mean±SEM; n=8 [R6/1-vehicle], n=8 [R6/1-RGFP109]; 8 striatal images were counted per animal. ns-not significant by unpaired Student's t-test. (B) Immunoblots showing H3K27ac or H3 patterns (each normalized to β-Actin levels) in protein extracts from striatal tissue. Data presented as mean±SEM; n=10 [WT-vehicle], n=12 [R6/1-vehicle], n=10 [WT-RGFP109], n=12 [R6/1-RGF109]; ns - not significant by two-way ANOVA with Tukey‘s multiple comparisons test.
