# Supplementary material for: The Effects of Selective Inhibition of Histone Deacetylase 1 and 3 in Huntington’s Disease Mice
Source: Front Mol Neurosci. 2021 Feb 17;14:616886. doi: 10.3389/fnmol.2021.616886 (PMC7925995; doi:10.3389/fnmol.2021.616886)
Supplement: Supplementary file 9 [file Table_1.pdf]

Table S1. Primer sequences

Gene		Sequence (5' → 3')
Actb	fw:	CACTGTCGAGTCGCGTCC
	rev:	TCATCCATGGCGAACTGGTG
Adora2a	fw:	GCCATCCCATTCGCCATCA
	rev:	GCAATAGCCAAGAGGCTGAAGA
Arc	fw:	TACCGTTAGCCCCTATGCCATC
	rev:	TGATATTGCTGAGCCTCAACTG
Cpne5	fw:	GTCTAACGGTGGTGTCCCAG
	rev:	TCCAGCTTGTTGGCACAGAA
Dpysl2	fw:	AAGCTGTGGTCACTGGGAAG
	rev:	GAAGACTTTGGCTGCGTTGG
Drd1	fw:	ATGGCTCCTAACACTTCTACCA
	rev:	GGGTATTCCCTAAGAGAGTGGAC
Drd2	fw:	AAGCGTCGGAAGCGGGTCAAC
	rev:	TCGGCGGGCAGCATCCATTCT
Egr1	fw:	TATGAGCACCTGACCACAGAGTCC
	rev:	CGAGTCGTTTGGCTGGGATAAC
Folr1	fw:	TTTACACGACCAGTGCAGCC
	rev:	TAGTTCCGCAGTGGTTCCAG
Grin3a	fw:	ACCAGTCAGAGGTTTCACAGAG
	rev:	GGTCCATCTTCTCCATCTGCTC
Nr4a2	fw:	GCAGAGAAGATCCCTGGCTT
	rev:	ACTGGGTTGGACCTGTATGC
Otx2	fw:	TCCAGGGTGCAGGTATGGTT
	rev:	AGCTCTTCTTCTTGGCAGGC
Polr2a	fw:	TGTGCAGGAAACATGACCGA
	rev:	GAAGCAGACACAGCGCAAAA
Ppp1r1b	fw:	CCAGAAACCCACTCTGTCCC
	rev:	GGCTTCAGCCAAAGCAAACA
Satb2	fw:	ACCGCACACAGGGATTATTGT
	rev:	CACTTCAGGCAGGTTGAGGA
Wars	fw:	AGCAGATCAAGAGCAAGGTCA
	rev:	TTCACAGTTGCCCCCAAACT
